# Intraluminal Administration of Poly I:C Causes an Enteropathy That Is Exacerbated by Administration of Oral Dietary Antigen

**DOI:** 10.1371/journal.pone.0099236

**Published:** 2014-06-10

**Authors:** Romina E. Araya, Jennifer Jury, Constanza Bondar, Elena F. Verdu, Fernando G. Chirdo

**Affiliations:** 1 Laboratorio de Investigación en el Sistema Inmune- LISIN, Departamento de Ciencias Biológicas, Facultad de Ciencias Exactas, Universidad Nacional de La Plata, La Plata, Argentina; 2 Division of Gastroenterology, Farncombe Family Digestive Health Institute, McMaster University, Hamilton, Canada; Cincinnati Children's Hospital Medical Center, University of Cincinnati College of Medicine, United States of America

## Abstract

Systemic administration of polyinosinic:polycytidylic acid (poly I:C), mimics virally-induced activation of TLR3 signalling causing acute small intestine damage, but whether and how mucosal administration of poly I:C causes enteropathy is less clear. Our aim was to investigate the inflammatory pathways elicited after intraluminal administration of poly I:C and determine acute and delayed consequences of this locally induced immune activation. Intraluminal poly I:C induced rapid mucosal immune activation in C57BL/6 mice involving IFNβ and the CXCL10/CXCR3 axis, that may drive inflammation towards a Th1 profile. Intraluminal poly I:C also caused enteropathy and gut dysfunction in gliadin-sensitive NOD-DQ8 mice, and this was prolonged by concomitant oral administration of gliadin. Our results indicate that small intestine pathology can be induced in mice by intraluminal administration of poly I:C and that this is exacerbated by subsequent oral delivery of a relevant dietary antigen.

## Introduction

Detection of components of microorganisms by a complex system of pattern recognition receptors leads to immune host defense. In the gut, home to 10^13^–10^14^ resident bacteria, but also fungi and viruses, the immune mechanisms elicited have a critical role in tissue homeostasis[1]. However, uncontrolled activation of these pathways may lead to pathology, as demonstrated in studies using intraperitoneal (ip) administration of double stranded RNA (dsRNA) that induces overactivation of TLR3 signalling [2], [3], [4], [5]. Polyinosinic:polycytidylic acid (poly I:C) is a synthetic analogue of dsRNA used to mimic the intermediates of replication present in cells infected with RNA viruses and some DNA viruses [6]. In addition to TLR3, dsRNA sensors include two cytoplasmic receptors, retinoic acid-inducible gene I (RIG-I) and melanoma differentiation-associated gene 5 (MDA5) [7]. NFκB and IRF3/7 are the dominant nuclear factors involved in the control of the expression of inflammatory cytokines and chemokines and Type I IFN, respectively, upon activation by dsRNA[8]. McAllister C. et al.^5^ showed that signaling pathways activated through dsRNA sensors and particularly, TLR3/TRIF/caspase 8, drive intestinal pathology but also host protection during viral infection. However, the role of locally induced, rather than systemic pathways in these models, remain unclear. More importantly there is a scarcity of models directly relevant to small intestinal induction of pathology and gut dysfunction.

Epidemiological studies support the association of enteric infections with the subsequent development of inflammatory or functional gastrointestinal disease [9], [10]. Viral infections, in particular enterovirus such as rotavirus, have been suggested to increase the incidence of celiac disease (CD), a permanent intolerance to gluten that occurs in genetically susceptible people [11], [12]. Case reports have also suggested the association of enteric infections with CD onset [13]. Currently, the underlying mechanisms that support this association are poorly understood. However, it is well known that innate immune activation generates a rapid and strong anti-viral response, which essentially involves the production of Type I IFNs and other components of the inflammatory response. The link among enterovirus infection and CD raises the hypothesis that Type I IFNs may be involved in loss of tolerance to gluten through the ability to promote inflammation and an strong Th1 response [14].

In this work, we aimed to mimic the local intestinal immune response as triggered by enteric viral infection. We used intraluminal administration of poly I:C to wild type mice and evaluated the immediate *in vivo* immune and pathological mechanisms triggered at the small intestinal mucosa. We also investigated the potential influence of this initial immune activation on long lasting gut functional responses to administration of a relevant dietary antigen. For this, we used NOD-DQ8 mice, a model that allows *in vivo* evaluation of functional and immune responses to gliadin, the storage protein in cereals [15].

Animal models rarely recapitulate the full spectrum of human complex diseases. Moreover, human diseases often express different clinical phenotypes. Thus the usefulness of animal models resides in mimicking certain aspects of disease pathogenesis in order to generate hypotheses to be tested in clinical trials. In this work, we used two animal models to investigate two different questions. First, the effect of oral poly I:C in the early stages of the mucosal innate immune response, and second the influence of the administration of a specific dietary antigen in preconditioned mice. We observed that luminal administration of poly I:C activated the innate immune response and caused acute enteropathy. Administration of gliadin to gliadin-sensitive mice previously treated with poly I:C, prolonged gliadin-induced gut dysfunction. We propose a mucosally-driven model of enteropathy by poly I:C in which post inflammatory mechanisms of gastrointestinal disease can be investigated.

## Materials and Methods

### Mice

Eight-week female C57BL/6J mice were purchased to the Animal Care Facility of the Faculty of Exact and Natural Sciences of the University of Buenos Aires. Mice were housed in plastic cages and fed *ad libitum* with balanced food and water. They were maintained on a 12h light/darkness cycle and acclimatized to the surrounding conditions for 1 week before the experimental procedure.

Transgenic mice that express HLA-DQ8 in an endogenous MHC class II-deficient background were backcrossed to NOD mice for 10 generations and intercrossed to produce congenic NOD AB° DQ8 mice [16]. Eight- to 10-wk-old male mice were used for experiments. Mice were weaned and maintained on a low-fat (4.4%), gluten-free diet. The original breeding pairs of NOD-DQ8 mice were kindly provided by Dr. Chella David and Dr. Joseph Murray from Mayo Clinic, Rochester, USA, and subsequently bred in specific pathogen-free conditions at McMaster University.

All the studies were performed in accordance with international protocols for laboratory animal care (Canadian Council on Animal Care). Experiments were conducted with approval from the McMaster University Animal Care Committee and the National University of La Plata Ethic Committee.

### Poly I:C treatment and oral gavage

Mice were kept on the gluten free diet at the time of the surgery. Poly I:C (Sigma-Aldrich) or PBS was intraluminally administrated throughout an intestinal microsurgery that allows inject the stimuli directly in the lumen of small intestine. Mice were anaesthetized with Ketamine 80 mg/kg of weight and Xilacine 10 mg/kg of weight. Once mice were asleep, 30μg/g of weight of poly I:C solution in PBS were injected in the lumen of small intestine, 2 cm after the stomach, to avoid the enzymatic degradation of the poly I:C (mainly because of nuclease activity from pancreatic enzymes). Control mice receipted only PBS in the lumen. After the surgery, a fluid replacement was done and mice were strictly monitored until they were able to walk around the cage.

A group of mice was sacrificed after 3h or 12h post-treatment with poly I:C or PBS, and samples of blood and small intestine were collected for further analysis.

Other group of mice was orally gavaged with 5 mg of α-gliadin/time, three times a week during 2 weeks, starting at 24h post-surgery. α-Gliadin (by Sigma-Aldrich) was dissolved in endotoxin-free acetic acid 0.02 M. Control mice were gavaged with the acetic acid solution only (Gluten-free diet mice). All solutions were controlled and endotoxin free preparations were determined using E-TOXATE endotoxin kit (Sigma). Mice were weighed three times a week during the 2 weeks, after the oral gavage.

### Histologic evaluation

Cross-sections of the proximal small intestine were fixed in 10% formalin, embedded in paraffin, and stained with H&E staining for histological evaluation by using a Nikon Eclipse Ti fluorescence microscope with X-Cites Series 120 Q light source. Images were taken with Nikon Digital Sight DS Ri1 camera using Nis-Elements software and measurements were performed using Image J software properly calibrated.

Two sections of the proximal small intestine were evaluated for evidence of inflammation. At least thirty villus-to-crypt ratios were measured for each mouse in a blinded fashion. Intraepithelial lymphocytes (IELs) per 30 enterocytes in ten randomly chosen villus tips were counted according to the previously described methods and expressed as IEL/100 enterocytes [17]. Histological scores were obtained from the small intestinal sections stained with H&E, following the Park-Chiu criteria[18]: 0, normal mucosa; 1, subepithelial space at villus tips; 2, extension of subepithelial space with moderate lifting; 3, massive lifting down sides of villi, some denuded tips; 4, denuded villi, dilated capillaries; 5, disintegration of *lamina propria*; 6, Crypt layer injury; 7, transmucosal infarction; 8, transmural infarction.

### Ussing chambers

Two sections of proximal small intestine from each NOD-DQ8 mouse were used for Ussing chamber experiments as described previously [15], [19], [20]. A 3–4-cm piece of small intestine was collected (3 cm from the stomach), cut into two sections, and placed in Krebs buffer aerated with 95% O_2_ and 5% CO_2_ (pH 7.3–7.4). Each segment of intestine was cut open along the mesenteric border to form a flat sheet and mounted into an Ussing chamber. The chamber exposed 0.7 cm^2^ of tissue surface area to 8 ml of circulating oxygenated Krebs buffer containing 10 mM glucose (serosal side) and 10 mM mannitol (mucosal side) maintained at 37°C. Net active ion transport across the epithelium was measured via a short circuit current (Isc, mA) injected across the tissue under voltage-clamp conditions. Tissue conductance (the passive permeability to ions) was calculated using Ohm's law. Baseline Isc (mA/cm2) and conductance (mS/cm^2^) were recorded at equilibrium, 20 min after mounting small intestine sections.

### Cytokine analysis

The presence of proinflammatory cytokines in serum from NOD-DQ8 mice was determined using a Cytometric Bead Array Inflammation kit (BD Biosciences) and analyzed using BD FACSarray Bioanalyzer System (BD Biosciences).

### Real Time PCR

Small Intestinal samples from C57BL/6 and NOD-DQ8 mice were stored in RNA later in-80°C freezer until be used. Tissues were disrupted and RNA extraction was performed using RNeasy Mini Kit (Qiagen). cDNA synthesis was performed from isolated RNA samples (2-5µg), using iScript Reverse Transcription Supermix (Bio-Rad). Real Time PCR reaction was performed with SsoFast EvaGreen Supermix (Bio-Rad) using the adequate forward and reverse primers and the iQ5 thermocycler with fluorescence detection (Bio-Rad). Reactions were run in triplicate. The Real Time PCR protocol was: Cycle 1 (1X) 95°C for 10min; Cycle2 (40X) 60°C for 1min and 95° for 15sec. Primers were synthesized as it was previously described [21]. The primer sequences are provided in Table 1. The geometric mean of housekeeping gene HPRT was used as an internal control to normalize the variability in expression levels. All results were expressed as a fold increase of PBS and PIC treatment versus the mean of PBS treatment in every time point (2^−ΔΔCt^ method).

**Table 1:** Primers used for quantitative PCR.

Gene	Forward	Reverse
**HPRT**	CAATGCAAACTTTGCTTTCC	CAAATCCAACAAAGTCTGGC
**MDA5**	CGATCCGAATGATTGATGCA	AGTTGGTCATTGCAACTGCT
**RIG-I**	CAGACAGATCCGAGACACTA	TGCAAGACCTTTGGCCAGTT
**TLR3**	GAAGATGATGCAGTCTTTCCA	CCTGTATCATATTCTACTCCTTGC
**IFNβ**	AATGGAAAGATCAACCTCAC	AAGGCAGTGTAACTCTTCTG
**CXCL10**	ATAGGGAAGCTTGAAATCATCC	TTCATCGTGGCAATGATCTC
**CXCR3**	TGTAGTTGGGCTAGCTCGAACTT	ACCTGGATATATGCTGAGCTGTCA
**TNFα**	CTCCCTCTCATCAGTTCTATGG	TTGAGAAGATGATCTGAGTGTG
**IL-15**	CATCCATCTCGTGCTACTTGTGTT	CATCTATCCAGTTGGCCTCTGTTT
**MCP1**	CTACAAGAGGATCACCAGCAG	TTCTGATCTCATTTGGTTCCG
**CXCL2**	AAGATACTGAACAAAGGCAAGG	TTCTTTCTCTTTGGTTCTTCCG
**IL-1β**	CGTTCCCATTAGACAACTGC	CTCATGGAGAATATCACTTGTTGG
**IL-18**	GATCAAAGTGCCAGTGAACC	GATCTTGTTCTTACAGGAGAGG
**IL-6**	CATGTTCTCTGGGAAATCGT	TATATCCAGTTTGGTAGCATCC
**IFNγ**	CTGAGACAATGAACGCTACAC	TTTCTTCCACATCTATGCCAC
**Bax**	TGCTACAGGGTTTCATCCAG	ATTGCTGTCCAGTTCATCTC
**Bcl2**	GATCTCTGGTTGGGATTCCT	ACAACTTGCAATGAATCGGG

### Fluorescence microscopy

Small intestinal sections from PBS- and PIC-treated mice were deparaffinized and an immunofluorescence protocol was performed. Antigenicity recovery was performed using Antigen Retrieval AR-10 Solution (BioGenex). After blocking with 2% goat serum, goat anti-IL-15 IgG antibody (R&D) was added in a concentration of 2 µg/ml for 1h. Alexa488 donkey anti-goat antibody (Molecular Probes) in a concentration of 10 µg/ml was added for 1h. Nuclei were stained with propidium iodide at 1 µg/ml for 15 minutes. Images were obtained and analyzed in a TCS SP5 Confocal Microscope combined with Leica LAS AF software.

### Statistical analysis

Statistical analysis was performed with GraphPad Prism software. For more than two treatment groups, an ANOVA with a Bonferroni post hoc test for multiple comparisons was used. When two groups were compared, an unpaired t test was used; p<0.05 was considered significant. Data are displayed as mean ±SEM.

## Results

### Intraluminal poly I:C induces histological changes in the small intestinal mucosa

**Figure 1:**
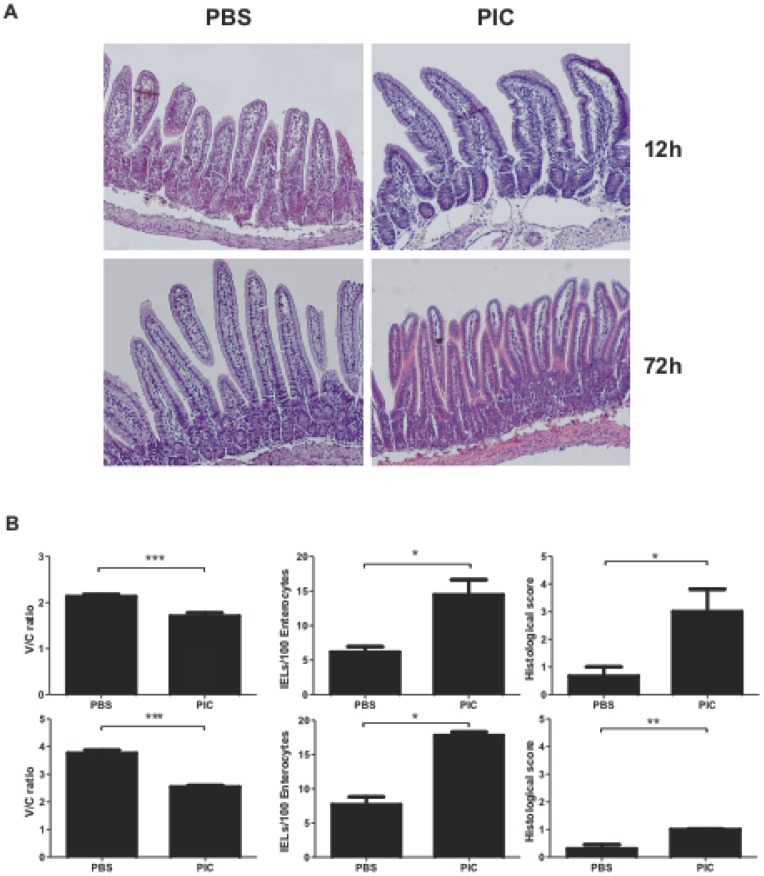
Intraluminal poly I:C induces enteropathy. Representative H&E stained sections of proximal small intestine of C57BL/6 mice after 12hs and 72hs of intraluminal administration of poly I:C or PBS (**A**). Morphological analysis of small intestine from C57BL/6 mice: villus-to-crypt (V/C) ratio, number of IELs and histological score after 12h (**B**) and 72h (**C**) (Stats: N =  4 mice per group, Unpaired t test, *P<0.05, **P<0.01, ***P<0.001).

We first evaluated the capacity of poly I:C delivered at the mucosal site to induce enteropathy in wild type mice. Intraluminal administration of poly I:C to C57BL/6 mice produced severe enteropathy. At 12h post poly I:C, we observed shortening and widening of villi, edema, and increased cell infiltration in the *lamina propria* (LP). Using H&E staining edema, dilated capillaries and interstitial spaces were observed in intestinal villi (**Figure 1A**). Furthermore, V/C ratios were decreased, while the number of IELs and global histological score increased compared to PBS-treated mice (**Figure 1B**). At 72h post poly I:C, there was partial recovery of villus-to-crypt (V/C) ratios. In contrast to control mice, morphometric parameters remained altered in poly I:C-treated mice (**Figure 1C**). As expected, the surgical procedure altered intestinal histology. However, this change was transient, and control mice exhibited a faster recovery after treatment. We also tested whether molecular weight of poly I:Cs influenced inflammation and mucosal damage. We did not detect significant differences among the tested poly I:Cs (**Figure S1**), and therefore the remaining experiments were carried out using the commonly used commercial poly I:C.

### Inflammatory mediators and receptors MDA5, RIGI and TLR3 are induced by intraluminal poly I:C in small intestine

**Figure 2:**
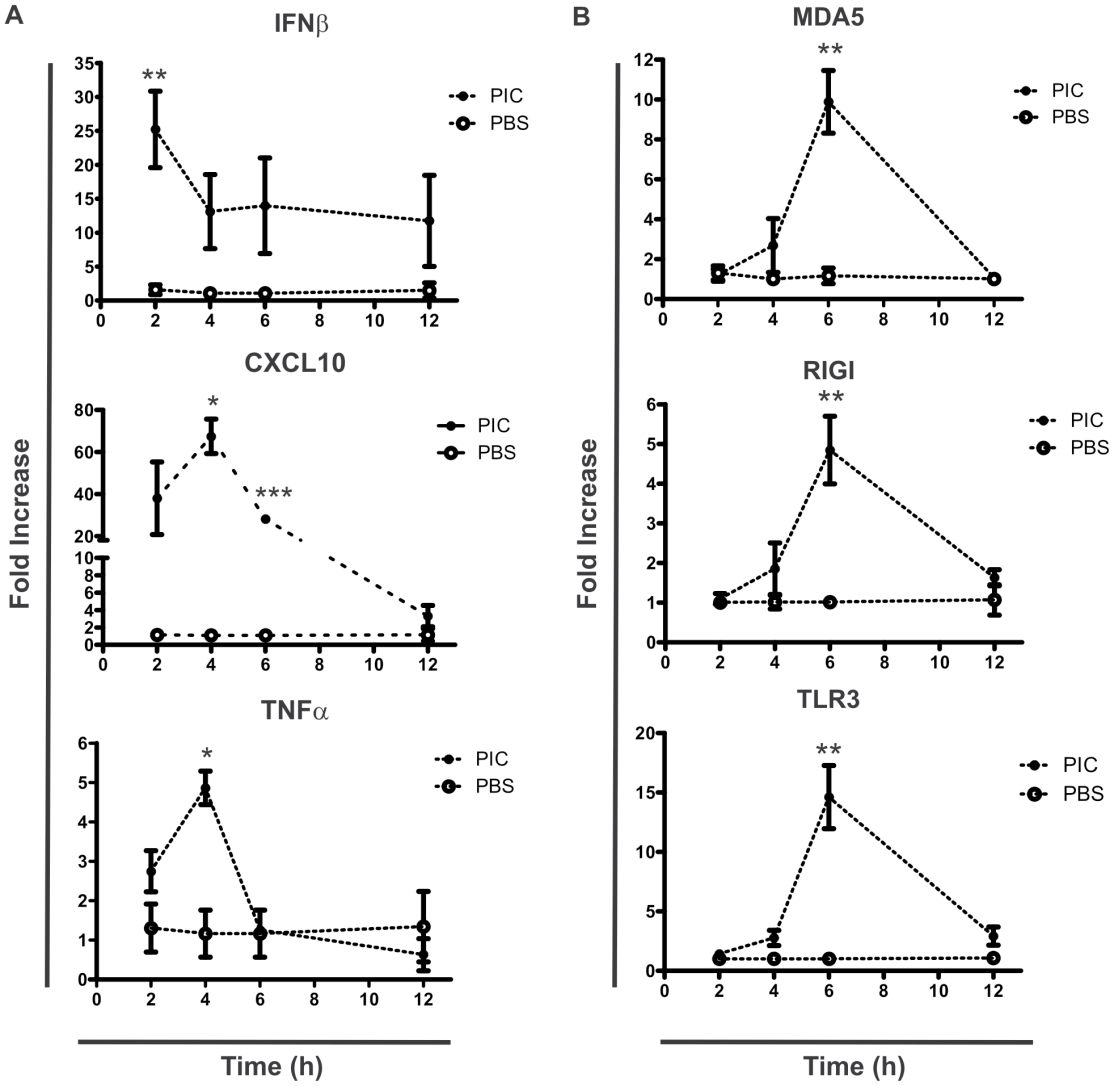
Intraluminal poly I:C generates a proinflammatory response and the induction of MDA5, RIG-I and TLR3 mRNA expression in small intestine. Real Time-PCR analysis of small intestinal samples from C57BL/6 mice after intraluminal administration of poly I:C or PBS was performed. Plots shows mRNA expression after 2 to 12h of poly I:C (black dots) or PBS (empty dots) treatment. IFNβ, CXCL10, and TNFα mRNA expression (A). MDA5, RIG-I, and TLR3 mRNA expression (B). All values were normalized with the housekeeping mRNA expression (HPRT). Results were expressed as fold increase of PBS and poly I:C treatment versus the mean of PBS treatment in every time point (2^-ΔΔCt^ method). Stats: N =  4 mice per group, Unpaired t test, *P<0.05, **P<0.01, ***P<0.001, poly I:C treated mice versus PBS control in the same time point.

We next explored the signaling pathways induced by intraluminal poly I:C. For this we analyzed the kinetics of expression of inflammatory mediators and three receptors for poly I:C in a time-course experiment using C57BL/6 mice. In the small intestine, there was a rapid and strong increment of IFNβ 2h after treatment, while CXCL10 and TNFα peaked 4h after intraluminal administration of poly I:C (**Figure 2A**). Analysis of expression of proinflammatory cytokines such as IL-18, IL-1β, IL-15 and IFNγ,chemokines such as MCP1 and CXCL2, and anti- and pro-apoptotic molecules Bcl-2 and Bax, respectively, did not show any differences between groups (**data not shown**).Since recognition of poly I:C is mediated by TLR3, MDA5 and RIGI and their expressions are modulated by poly I:C, we evaluated whether the expression of these receptors were induced in the small intestine of treated mice. The time course experiment showed that MDA5, RIGI and TLR3 mRNA were similarly upregulated in small intestine by intraluminal injection of poly I:C. Expression of the three receptors peaked 6h post-treatment (**Figure 2B**). Furthermore, we analyzed the protein expression of IL-15, obtaining no relevant differences among poly I:C and PBS treatments after 12h (**data not shown**).

### Inflammatory response induced by intraluminal poly I:C in the small intestine of NOD-DQ8 mice

**Figure 3:**
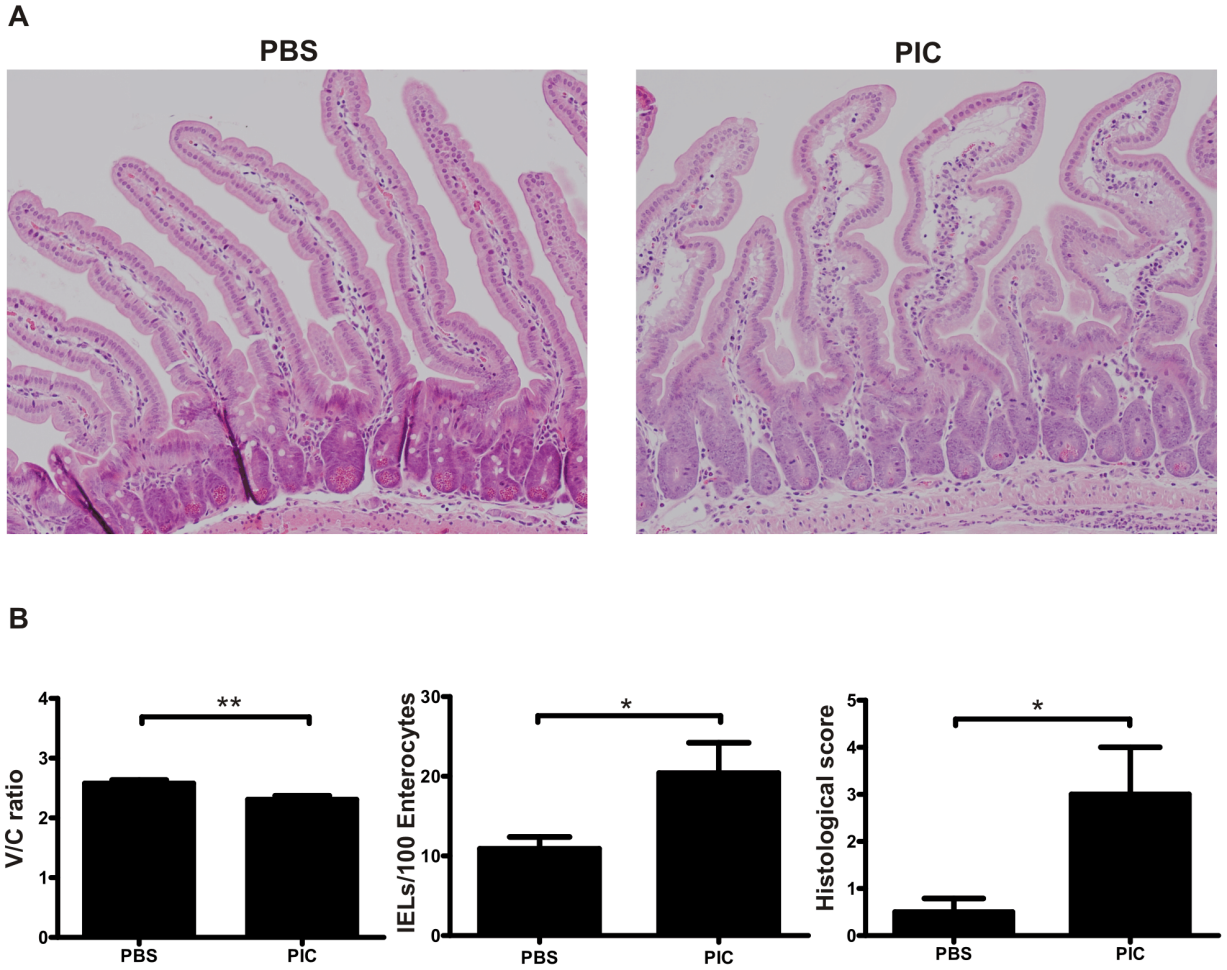
Intraluminal administration of poly I:C induces enteropathy in NOD-DQ8 mice. Representative H&E-stained sections of the proximal small intestine in PBS and poly I:C-treated NOD-DQ8 mice at 12h post-treatment (**A**). Morphological analysis of small intestine from NOD-DQ8 mice: villus-to-crypt (V/C) ratio, number of IELs and histological score after 12h. Stats: N =  4 mice per group, Unpaired t test, *P<0.05, **P<0.01 (**B**).

The NOD-DQ8 mouse represents one useful model of gluten sensitivity [15]. Nevertheless, the model has particular phenotypes given by the DQ8 restriction and the NOD background [22]. Thus, prior to gliadin antigen administration, we first examined in detail the immune responses to poly I:C in NOD-DQ8 mice. Indeed, poly I:C caused severe mucosal damage in NOD-DQ8 mice characterized by cell infiltration of the LP, edema and shortening of villi at 12h post-treatment (**Figure 3A**). Morphometric parameters revealed marked reduction in the V/C ratio in poly I:C treated NOD-DQ8 mice. Also, IELs numbers and histological scores were higher in poly I:C-treated mice than in control NOD-DQ8 mice (**Figure 3B**).

**Figure 4:**
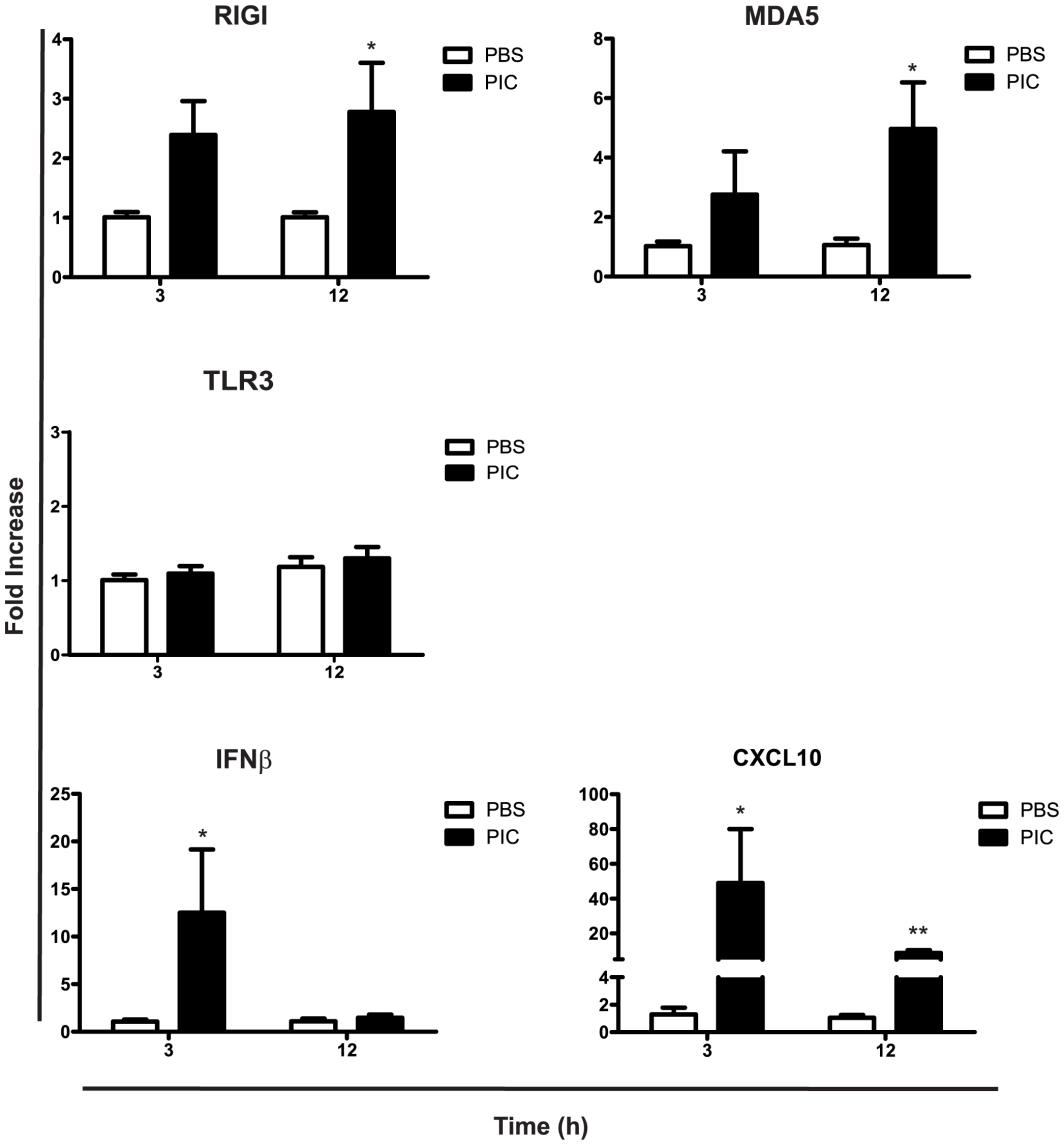
Poly I:C generates a proinflammatory response and induces RIG-I and MDA5 mRNA expression in the small intestine from NOD-DQ8 mice. Real Time-PCR analysis of the IFNβ, CXCL10, TLR3, MDA5, and RIG-I mRNA expression at 3 and 12 h post-treatment with poly I:C (black bar) or PBS (white bar). All values were normalized to the housekeeping mRNA expression (HPRT). All results were expressed as a fold increase of PBS and poly I:C treatment versus the mean of PBS treatment in every time point (2^−ΔΔCt^). Stats: N =  4 mice per group, Unpaired t test, *P<0.05, **P<0.01, poly I:C-treated mice versus PBS control in the same time point.

**Figure 5:**
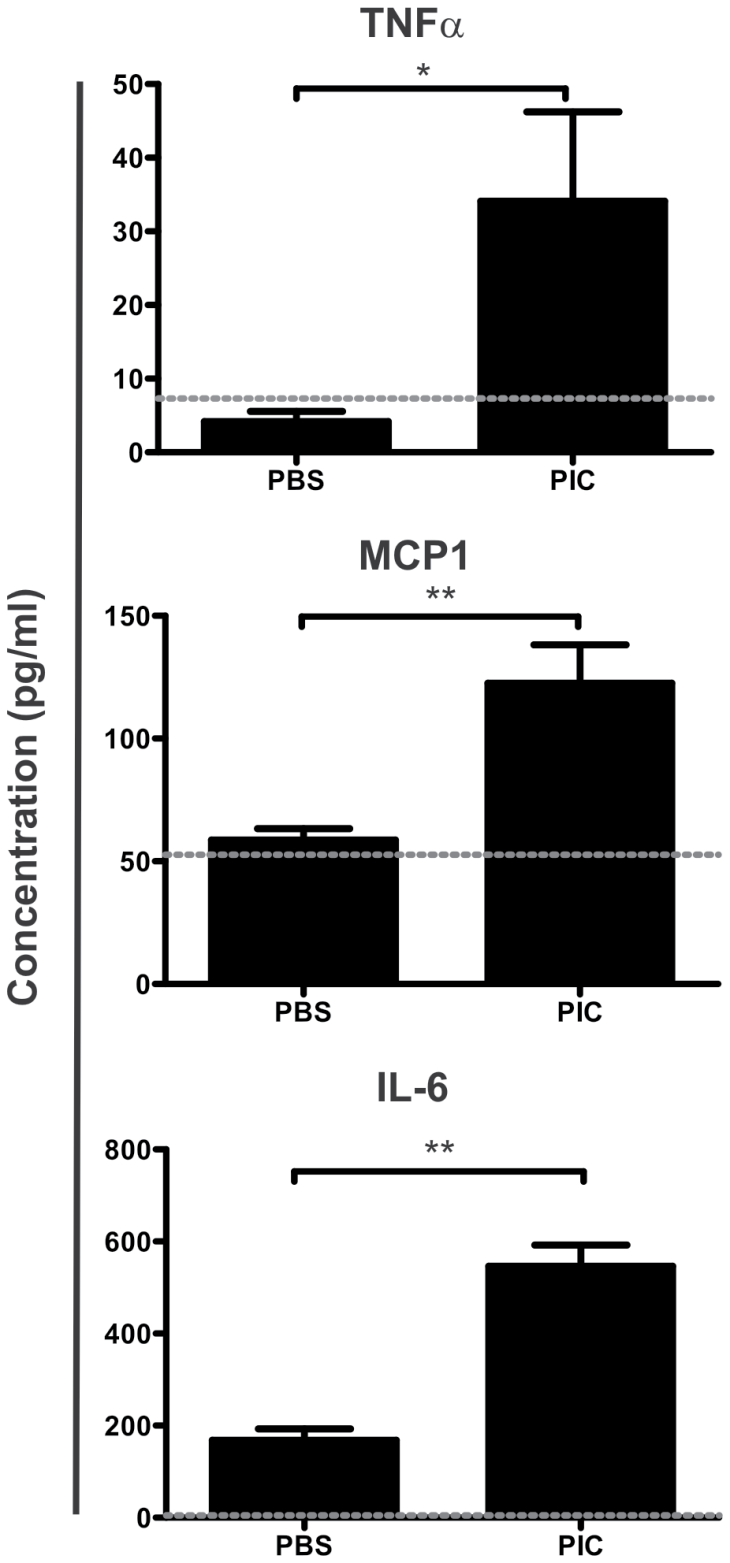
Increased serum levels of TNFα, MCP-1, and IL-6 after intraluminal poly I:C administration. TNFα, MCP-1, and IL-6 concentration was assessed in serum after 3h of poly I:C or PBS treatment, using a cytometric bead array inflammation kit. The dotted line represents the limit of detection. Stats: N =  4 mice per group, Unpaired t test, *P<0.05, **P<0.01.

We found that IFNβ mRNA expression was increased in the small intestinal mucosa of NOD-DQ8 mice at 3h post-poly I:C compared to controls (**Figure 4**). A 40-fold increment in CXCL10 mRNA was observed at 3 h in poly I:C-treated mice. At 12 hours, CXCL10 levels remained higher in poly I:C-treated mice than in controls. The cytoplasmic pattern recognition receptors for poly I:C, MDA5 and RIG-I, were also induced in the small mucosa of poly I:C-treated NOD-DQ8 mice. Expression of both receptors was increased at 3h, but was higher at 12 h after treatment. The upregulation of these receptors was observed later than in C57BL/6 mice. At 3h and 12h post-Poly I:C injection we did not observe differences in TLR3 expression when compared to PBS controls. Inflammatory mediators elicited by intraluminal poly I:C were also evaluated in peripheral blood. Concentration of TNFα, IL-6 and MCP1 was significantly higher 3h after intraluminal administration of poly I:C than in controls (**Figure 5**), while IL-10, IL-12p70 and IFNγ did not show changes in poly I:C-stimulated mice versus PBS controls (**data not shown**). Altogether these results suggest a systemic pro-inflammatory state.

### Intraluminal poly I:C and oral dietary antigen induce prolonged enteropathy in NOD-DQ8 mice

**Figure 6:**
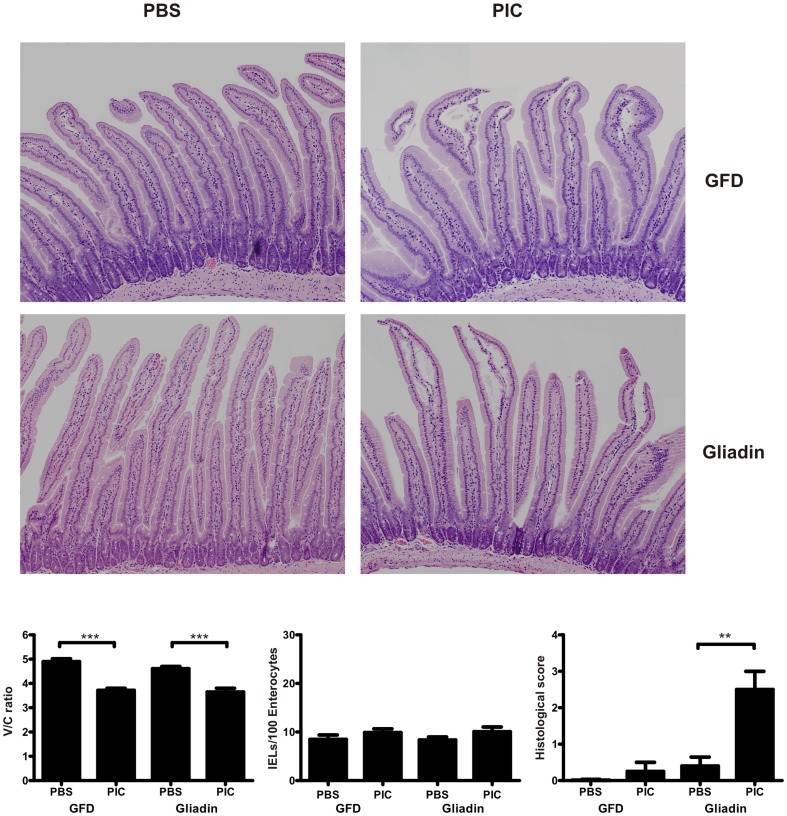
Gliadin induces histological damage in intraluminal poly I:C-treated NOD-DQ8 mice. Representative H&E-stained sections of the proximal small intestine from PBS and poly I:C-treated NOD-DQ8 mice on a gluten free diet (GFD) or orally gavaged with gliadin during 2 weeks (**A**). Morphological analysis of small intestine from NOD-DQ8 mice treated with poly I:C or PBS under a gluten free diet (GFD) or gavaged with gliadin during 2 weeks: V/C ratio, number of IELs and histological score. Stats: N =  4-5 mice per group, Unpaired t test, **P<0.01, ***P<0.001 (**B**).

Once we had determined the local and systemic immune responses of NOD-DQ8 mice to mucosally-administered poly I:C, we evaluated whether this was potentiated or prolonged by oral administration of relevant dietary antigen. NOD-DQ8 mice were treated with poly I:C and fed gliadin or a gluten-free chow, starting 24h later, three times a week during two weeks. Small intestinal sections and blood samples were collected after two weeks. A gluten-free chow did not affect the level of enteropathy in NOD-DQ8 mice induced by intraluminal poly I:C. H&E staining showed subepithelial edema and moderate lifting of villi in poly I:C-treated mice with or without gliadin (**Figure 6A**).Histological parameters revealed that both groups had an altered V/C ratio, but similar IELs counts, 2 weeks after poly I:C administration (**Figure 6B**). However, persistent morphological changes in the small intestinal mucosa were only observed in poly I:C-treated NOD-DQ8 mice that were subsequently challenged with gliadin, indicating maintenance of intestinal damage by oral antigen administration.

**Figure 7:**
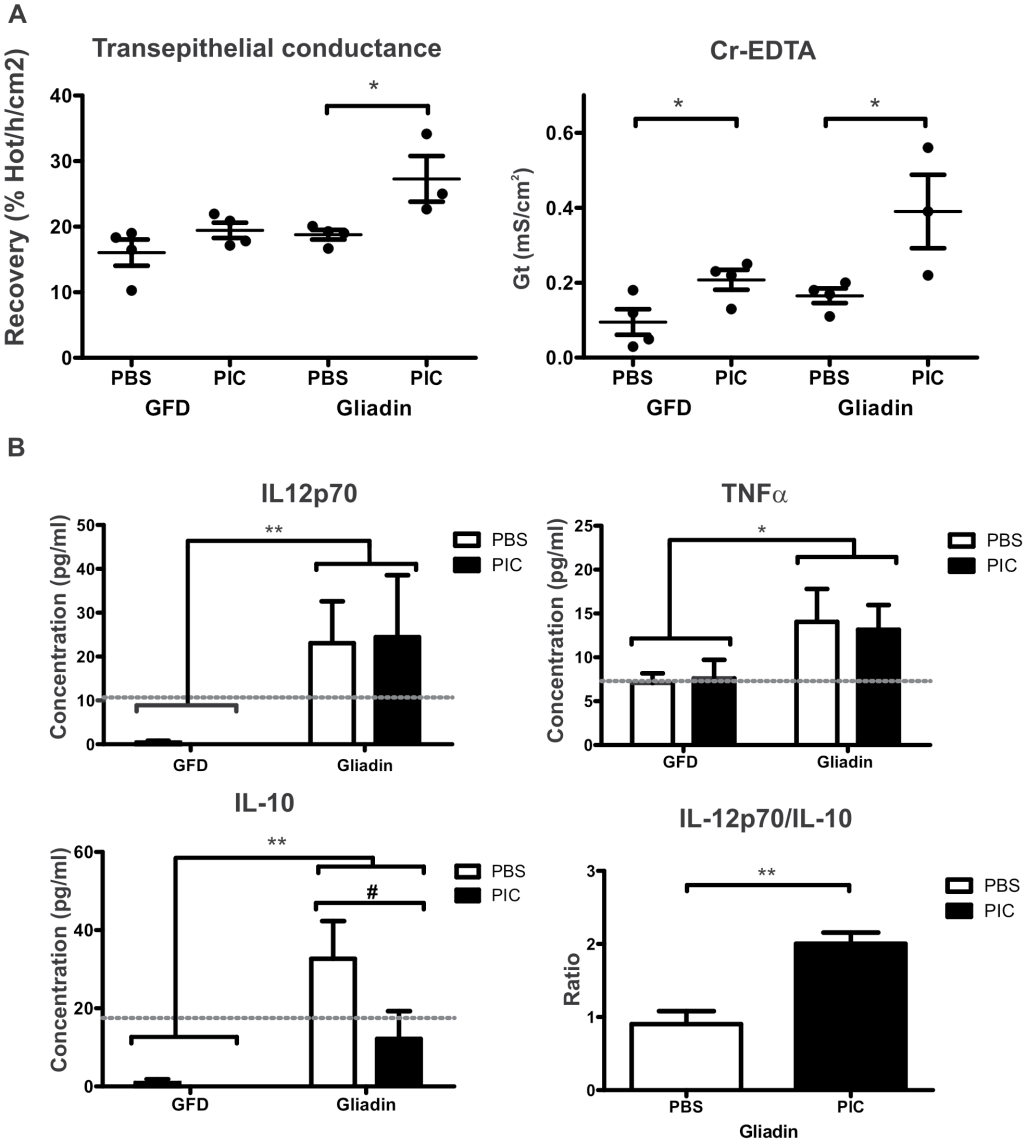
Gliadin induces barrier dysfunction and proinflammatory cytokines in poly I:C-treated NOD-DQ8 mice. After 24h of the final gavage, sections of small intestine were mounted in Ussing chambers and tissue conductance (mS/cm2) and isotopic flux (% Hot/h/cm^2^) were measured. Each dot represents an individual mouse and is a mean of 2 independent tissues. Stats: N =  3-4 mice per group, Two way ANOVA and Bonferroni post-test, *P<0.05 (**A**). Analysis of IL-12p70, TNFα and IL-10 in serum samples from poly I:C or PBS-treated mice on a gluten-free diet (GFD) or gavaged with gliadin for 2 weeks. IL-12p70/IL-10 ratio was determined. Serum concentration of cytokines was determined by a cytometric bead array inflammation kit. The dotted line represents the limit of detection. Stats: N =  4-5 mice per group, Two way ANOVA and Bonferroni post-test, *P<0.05, **P<0.01 gliadin versus GFD mice; #P<0.05 poly I:C versus PBS in Gliadin-gavaged mice (**B**).

We also evaluated intestinal barrier function by Ussing chamber technique. Oral gavage of gliadin for two weeks was associated with increased transepithelial conductance in poly I:C-treated NOD-DQ8 mice. This change was not observed in poly I:C-treated mice fed a gluten free chow or in control mice (**Figure 7A**).The evaluation of isotopic flux of ^51^Cr-EDTA showed increased paracellular permeability two weeks after poly I:C treatment regardless of the diet. However the percentage increase was higher in mice receiving gliadin gavages than in those on gluten-free chow (mean value for each treatment: PBS-GFD: 0.095%, poly I:C-GFD: 0.22%, PBS-gliadin: 0,17%, poly I:C-Gliadin: 0,56% Hot/h/cm^2^). The levels of the proinflammatory cytokines IL-12p70 and TNFα were increased in peripheral blood of mice gavaged with gliadin after 2 weeks compared with those on gluten free chow, even in the absence of poly I:C treatment (**Figure 7B**). However no differences were observed in MCP-1, IL-6 and IFNγ cytokine production in any of groups (**data not shown**). Gliadin gavages also induced an increment in the anti-inflammatory cytokine IL-10 in serum. Remarkably, poly I:C treatment abolished the production of IL-10 induced by gliadin. Therefore, orally delivered gliadin in poly I:C treated mice produced a substantial imbalance in the IL-12p70/IL-10 ratio, suggesting a long lasting proinflammatory milieu. Serum levels of IL-12p70 and IL-10 were below the limit of detection in mice on gluten-free chow. These results suggest that local inflammatory and functional processes in the small intestine induced by intraluminal poly I:C predispose to stronger functional impairment and inflammatory response to a relevant orally-administered dietary antigen.

## Discussion

We showed that induction of local small intestinal inflammation can be elicited by intraluminal administration of poly I:C to both wild type and gliadin-sensitive NOD-DQ8 mice. We determined that the severe enteropathy induced by intraluminal poly I:C, predisposed to functional impairment of small intestine in NOD-DQ8 mice receiving the dietary antigen, gliadin.

During viral enteric infections, intermediates of replication, such as dsRNA, are produced in infected cells [6] and trigger a rapid inflammatory cascade, which involves the activation of TLR3 and RNA helicases such as RIG-I and MDA5 [7]. Poly I:C is a synthetic analogue commonly used to mimic the effects of dsRNA which causes intestinal pathology when injected parentally [2], [3], [4]. The marked villus shortening and increased enterocyte apoptosis observed after parenteral poly I:C has been suggested to be caused by TLR3/TRIF/Caspase 8 activation[5]. We used instead intraluminal administration of poly I:C and demonstrated it produced severe intestinal pathology, as assessed by reduced V/C ratio, increased IELs counts and increased global histological scores. Induction of dsRNA sensors TLR3, MDA5 and RIG-I was transient, peaked at 6h and returned to basal levels at 12h. In previous models using parenteral poly I:C administration, mucosal damage seemed to be specific for TLR3 activation[3], [4], [5]. The underlying mechanisms proposed for intestinal damage include NK cells [2] and CD8αα^+^ IELs with critical participation of IL-15 [3], [4]. A recent study however has proposed that, neither NK cells, IELs or IL-15 are involved in the induction of intestinal pathology [5]. Using intraluminal poly I:C, we found a strong and early increase of IFNβ mRNA levels in the small intestine of C57BL/6 mice 2h post-treatment, indicating a mucosal specific poly I:C-signaling activation. TNFα, a cytokine commonly involved in the early inflammatory response, was also transiently upregulated. Remarkably, a 60-fold increase in CXCL10 expression was observed 4h after intraluminal poly I:C. CXCL10 has previously been shown to be upregulated by poly I:C in different experimental strategies[23], and it may play a crucial role in the development of small intestine pathology since it is a major chemo-attractant for Th1 CXCR3^+^ cells into inflamed tissues [24], [25]. The mRNA expression of proinflammatory cytokines such as IL-15, IL-18, IL-1β and IFNγ, chemokines such as MCP1 and CXCL2 and anti- and pro-apoptotic molecules as Bcl-2 and Bax respectively, were analyzed, but no changes were observed (**data not shown**). This is consistent with the report by McAllister C. et al. [5] using a parenteral poly I:C model.

Since poly I:C triggers a cascade of inflammatory reactions involving different signaling pathways some of the effects after poly I:C administration may also have diverse origins. The observation that transglutaminase 2 (TG2) is activated in small intestine by intraperitoneal poly I:C administration highlights that different players may contribute to additional dysregulation of gut homeostasis[26]. Upregulation of TG2 has also been related to NFkB pathway, driving an amplification loop of inflammation and damage at the small intestinal mucosa[27]. Overall our results suggest that intraluminal poly I:C induces rapid mucosal immune activation involving IFNβ, and the CXCL10/CXCR3 axis that may drive inflammation towards a Th1 profile.

Understanding the early and delayed consequences of immune activation by enteric viral infections, or dsRNA, has important implications for post-infectious inflammatory and functional consequences [10]. Thus our second aim was to determine whether a single intraluminal administration of poly I:C influenced subsequent responses to gliadin, a dietary antigen that causes CD in genetically susceptible people, but which may be involved in gut functional abnormalities in a subgroup of patients with irritable bowel syndrome (non celiac gluten sensitivity) [28]. For this we used NOD-DQ8 mice, which lack all endogenous class II molecules and express the human HLA-DQ8 gene involved in CD [16], [29]. Previous studies using this model have shown that gliadin sensitization results in barrier dysfunction and moderate enteropathy [15]. Poly I:C-treated NOD-DQ8 mice developed mucosal damage characterized by higher histological score than in control mice, cell infiltration in LP, reduction of V/C ratio, increased IEL counts, and upregulation of IFNβ and CXCL10 mRNA in intestinal mucosa. This was also accompanied by higher levels of TNFα, IL-6 and MCP1 in peripheral blood. After poly I:C administration, one group of NOD-DQ8 mice was subjected to oral gliadin challenges while another was fed a gluten free chow. ^51^Cr-EDTA flux studies indicated increased paracellular permeability in poly I:C-treated mice after two weeks regardless of the dietary treatments. Poly I:C also induced alterations in V/C ratio compared to control mice in both gliadin challenged and gluten free mice. However, only poly I:C-treated mice receiving gliadin presented higher global histological scores, demonstrating severe changes in the mucosa. More importantly, increased transepithelial conductance was only observed in poly I:C-treated NOD-DQ8 mice that were subsequently challenged with gliadin. Remarkably, after two weeks of gliadin ingestion, NOD-DQ8 mice showed increased levels of the proinflammatory cytokines IL-12p70 and TNFα in peripheral blood, compared with those on gluten-free chow, indicating a causal link to gliadin challenges and not to poly I:C treatment. Moreover, IL-10 induction by gliadin, a feature observed in -DQ8 mouse models of gluten sensitivity before [15], [30], [31], was inhibited in previously poly I:C-treated mice. An imbalance in the IL-12p70/IL-10 ratio was observed in gliadin challenged and poly I:C-treated NOD-DQ8 mice. The inflammatory effects elicited by IL-12p70 and TNFα have been shown to have direct effects on barrier function and intestinal damage. The correct assembly of tight junction components is critical to preserve barrier integrity and TNFα is recognised as one of the main mediators associated with inflammation in Crohn's disease and CD. TNFα signalling activates myosin light chain kinase (MLCK) and tight junction remodelling leading to loss of barrier function [32], [33]. It is noteworthy that gliadin peptides can also increase permeability by innate mechanisms amplifying the circuit of mucosal damage [34], [35], [36], [37].

We did not observe changes in IL-15 expression associated with this model. IL-15 expression has a complex post-transcriptional control which is particularly important to maintain an adequate balance of the immune response in gut. Remarkably, IL-15 expression has been shown to be modulated by p31-43 gliadin peptide, which triggers different innate responses in Caco-2 cells as well as in human duodenum [38]. Though we did not specifically work with p31-43, we have performed significant characterization of the NOD-DQ8 model [15].Given the NOD background of mice this model is not specifically IL-15 driven. Thus, neither mRNA nor protein IL-15 determinations in this particular model are useful markers.

In conclusion, intraluminal poly I:C induced a severe but transitory damage in the small intestinal mucosa in both wild-type and NOD-DQ8 mice.The combination of poly I:C and subsequent gliadin feeding in the genetically susceptible mouse strain NOD-DQ8, led to a proinflammatory synergistic effect and impairment of gut barrier function. Our results raise the intriguing hypothesis thattype I IFNs constitute an important contributor to the loss of tolerance to dietary antigens such as gluten, after a viral infection. This may be particularly important in genetically susceptible hosts, by promoting inflammation as well as a biased Th1 response. We hypothesize that this may provide a mechanistic explanation for the clinical link between enteric infections and post-inflammatory and functional disorders [11], [12], [39], [40], [41].

## Supporting Information

Figure S1 Different molecular weight poly I:Cs cause enteropathy. Analysis of different molecular weight poly I:Cs by ethidium bromide-stained agarose gel electrophoresis, commercial poly I:C (PICs) (Sigma-Aldrich), PICL (LMW-PIC) and PICH (HMW-PIC) (Invivogen) (**A**). Morphological analysis performed in C57BL/6 mice intraluminally treated with PICs, PICL or PICH in a time-course experiment (from 12 up to 72hs) (**B**): V/C ratio vs. time (**i**); V/C ratio at 72h post-treatment with different poly I:Cs or PBS (**ii**); Histological score at 12h and 72h after different poly I:Cs treatments (**iii**). (Stats: N =  4 mice per group, Unpaired t test, *P<0.05, **P<0.01, ***P<0.001).
(TIF)
