# Supplementary figures and images for: Intraluminal Administration of Poly I:C Causes an Enteropathy That Is Exacerbated by Administration of Oral Dietary Antigen
Source: PLoS One. 2014 Jun 10;9(6):e99236. doi: 10.1371/journal.pone.0099236 (PMC4051664; doi:10.1371/journal.pone.0099236)

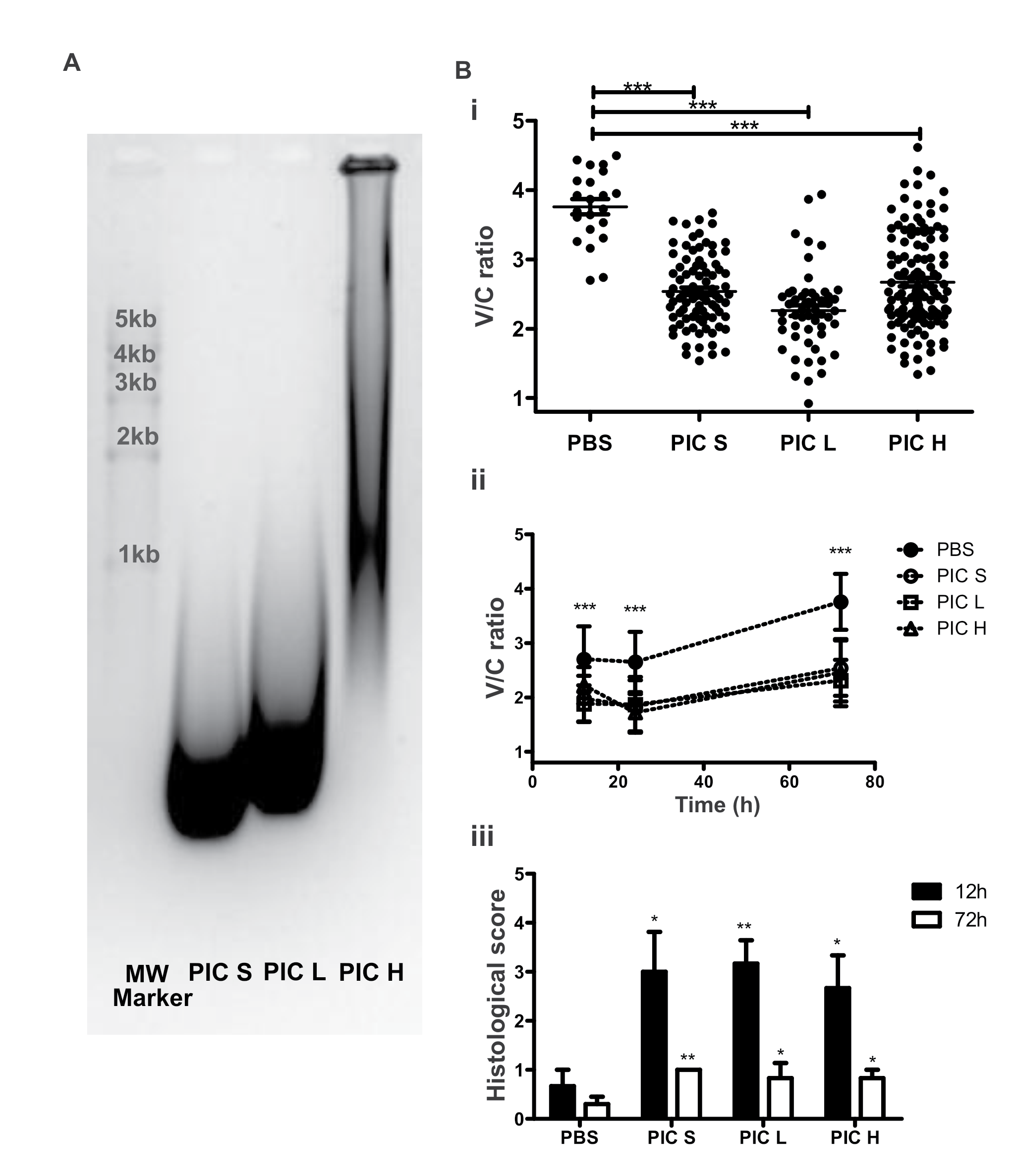

Supplement: Figure S1 — Different molecular weight poly I:Cs cause enteropathy. Analysis of different molecular weight poly I:Cs by ethidium bromide-stained agarose gel electrophoresis, commercial poly I:C (PICs) (Sigma-Aldrich), PICL (LMW-PIC) and PICH (HMW-PIC) (Invivogen) (A). Morphological analysis performed in C57BL/6 mice intraluminally treated with PICs, PICL or PICH in a time-course experiment (from 12 up to 72hs) (B): V/C ratio vs. time (i); V/C ratio at 72h post-treatment with different poly I:Cs or PBS (ii); Histological score at 12h and 72h after different poly I:Cs treatments (iii). (Stats: N = 4 mice per group, Unpaired t test, *P<0.05, **P<0.01, ***P<0.001). (TIF) [file pone.0099236.s001.tif]
